# A method for the generation of pseudovirus particles bearing SARS coronavirus spike protein in high yields

**DOI:** 10.1247/csf.21047

**Published:** 2022-04-28

**Authors:** Yoichiro Fujioka, Sayaka Kashiwagi, Aiko Yoshida, Aya O. Satoh, Mari Fujioka, Maho Amano, Yohei Yamauchi, Yusuke Ohba

**Affiliations:** 1 Department of Cell Physiology, Faculty of Medicine and Graduate School of Medicine, Hokkaido University, N15W7, Kita-ku, Sapporo 060-8638, Japan; 2 Global Station for Biosurfaces and Drug Discovery, Hokkaido University, N12W6, Kita-ku, Sapporo 060-8612, Japan; 3 AMED-CREST, Japan Agency for Medical Research and Development, N15W7, Kita-ku, Sapporo 060-8638, Japan; 4 School of Cellular and Molecular Medicine, University of Bristol, University Walk, Bristol BS8 1TD, UK; 5 Division of Biological Science, Graduate School of Science, Nagoya University, Furo-cho, Chikusa-ku, Nagoya, 464-8601, Japan

**Keywords:** severe acute respiratory syndrome coronavirus (SARS-CoV), SARS-CoV-2, pseudovirus, vesicular stomatitis virus (VSV), spike protein

## Abstract

The ongoing severe acute respiratory syndrome coronavirus 2 (SARS-CoV-2) pandemic has threatened human health and the global economy. Development of additional vaccines and therapeutics is urgently required, but such development with live virus must be conducted with biosafety level 3 confinement. Pseudotyped viruses have been widely adopted for studies of virus entry and pharmaceutical development to overcome this restriction. Here we describe a modified protocol to generate vesicular stomatitis virus (VSV) pseudotyped with SARS-CoV or SARS-CoV-2 spike protein in high yield. We found that a large proportion of pseudovirions produced with the conventional transient expression system lacked coronavirus spike protein at their surface as a result of inhibition of parental VSV infection by overexpression of this protein. Establishment of stable cell lines with an optimal expression level of coronavirus spike protein allowed the efficient production of progeny pseudoviruses decorated with spike protein. This improved VSV pseudovirus production method should facilitate studies of coronavirus entry and development of antiviral agents.

## Introduction

Severe acute respiratory syndrome coronavirus 2 (SARS-CoV-2) is an enveloped, positive-strand RNA virus that belongs to the *Coronaviridae* family and is responsible for the recent pandemic of coronavirus disease 2019 (COVID-19). As of 15 June 2021, there had been >174 million confirmed cases of and ~3.7 million deaths from COVID-19 reported in over 220 countries, areas, or territories (https://www.who.int/emergencies/diseases/novel-coronavirus-2019).

The envelope of SARS-CoV-2, like that of SARS-CoV, contains three structural proteins: the spike glycoprotein (hereafter referred to simply as S protein), membrane protein, and small envelope protein (Schoeman and Fielding, 2019; Wrapp *et al.*, 2020). Together with retrovirus envelope proteins and influenza virus hemagglutinin proteins, S protein is categorized as a class I viral fusion protein (Millet and Whittaker, 2018; White *et al.*, 2008), and it is targeted to the rough endoplasmic reticulum (rER) of host cells by an NH_2_-terminal signal sequence (Breitling and Aebi, 2013; Duan *et al.*, 2020; Lontok *et al.*, 2004; Sadasivan *et al.*, 2017). Cleavage of the signal sequence is followed by resumption of peptide chain elongation and insertion of the synthesized S protein into the ER membrane as a homotrimer (Binns *et al.*, 1985; Braakman and Hebert, 2013; Delmas and Laude, 1990; Duan *et al.*, 2020). S protein plays a key role in both specific interaction of the virus with its host cell receptor and subsequent virus internalization via endocytosis or membrane fusion. Binding of the coronavirus to its host cell receptor, angiotensin-converting enzyme 2 (ACE2), has been attributed to the S1 receptor-binding subunit region of S protein, which adopts an active “standing-up” conformation for receptor binding and an inactive “lying-down” conformation for immune evasion (Guan *et al.*, 2020; Wrapp *et al.*, 2020). After the S1 subunit region binds to ACE2, S protein undergoes proteolytic cleavage into S1 and S2 fusion subunits at the S1/S2 site in the presence of transmembrane protease serine 2 (TMPRSS2) and furin (Bestle *et al.*, 2020; Jaimes *et al.*, 2020). Additional cleavage sites located within the S2 subunit are cleaved by host cell proteases including cathepsins, furin-like proprotein convertases, and trypsin-like serine protease (Burkard *et al.*, 2014; Coutard *et al.*, 2020; Hoffmann *et al.*, 2020). The exposed S2 subunit mediates membrane fusion either at the plasma membrane or with the endosomal membrane, depending on protease availability in the host cell. Characterization of S protein is thus crucial for a better understanding of SARS-CoV-2 entry, but such studies are often limited because experiments with the live virus must be conducted in a biosafety level 3 (BSL-3) containment facility.

Pseudotyped virus systems are highly useful for characterization of viral envelope proteins and investigation of their role in virus entry (de Vries *et al.*, 2011; Hoffmann *et al.*, 2020; Johnson *et al.*, 2020; Kalkeri *et al.*, 2021; Miyauchi *et al.*, 2009; Nanbo *et al.*, 2010; Rentsch and Zimmer, 2011; Schelhaas *et al.*, 2012). Virus particles produced with such a system consist of a surrogate viral core and heterologous viral envelope proteins. The genome of the parental virus is modified to remove essential genes for viral reproduction, so as to prevent the generation of infectious progeny viruses. This approach allows viruses pseudotyped with envelope proteins of highly pathogenic viruses to be handled safely in a BSL-2 containment facility instead of a BSL-3 or BSL-4 facility. Vesicular stomatitis virus (VSV), a negative-strand RNA virus of the *Rhabdoviridae* family, has been widely adopted in both pseudotyped and recombinant virus systems (Hoffmann *et al.*, 2020; Johnson *et al.*, 2020; Kalkeri *et al.*, 2021; Miyauchi *et al.*, 2009; Nanbo *et al.*, 2010; Rentsch and Zimmer, 2011). It possesses five structural proteins—the glycoprotein (G), large polymerase protein (L), matrix protein (M), nucleoprotein (N), and phosphoprotein (P) (Lichty *et al.*, 2004)—among which G protein contributes to binding to the host cell surface and fusion with the endosomal membrane. VSV was the first negative-strand RNA virus for which a reverse genetics approach was established (Lawson *et al.*, 1995), an approach that allowed packaging of a reporter gene into the viral genome for quantitative evaluation of infectivity of recombinant viruses on the basis of the reporter gene activity. It was also found that a large amount of morphologically intact virus particles could be produced even in the absence of G protein. In addition, coinfection of cells with VSV and other viruses results in the formation of VSV pseudotyped with heterologous envelope proteins (Huang *et al.*, 1974; Witte and Baltimore, 1977). With these features, VSV-based pseudotyping is a potentially powerful tool for studies of the role of envelope proteins, including S proteins of SARS-CoV and SARS-CoV-2, in virus entry (Clausen *et al.*, 2020; Hoffmann *et al.*, 2020).

During the course of experiments with VSV pseudoviruses bearing S protein of SARS-CoV or SARS-CoV-2 described here, we found that most pseudotyped particles produced by the conventional transfection protocol lack S protein, likely because S protein expression at a high level in the virus-producing cells suppresses infection by parental VSV. We therefore developed an improved protocol based on cell lines stably expressing S proteins. This new method generates VSV pseudotyped particles decorated with S protein at high efficiency and with high infectious yields compared to the conventional approach. Our protocol increases the fidelity of SARS-CoV-2 entry research and should facilitate the development of new antiviral agents.

## Materials and Methods

### Cell lines

HEK293T (ATCC CRL-11268), VeroE6 (ATCC CRL-1586), and BEAS-2B (ATCC CRL-9609) cells were obtained from American Type Culture Collection (ATCC; Manassas, VA, USA), and VeroE6/TMPRSS2 (JCRB1819) cells were from the Japanese Collection of Research Bioresources cell bank (JCRB; Osaka, Japan). These cells were maintained under a humidified atmosphere of 5% CO_2_ at 37°C in Dulbecco’s modified Eagle’s medium (DMEM) (Sigma-Aldrich, Tokyo, Japan) supplemented with 10% fetal bovine serum (FBS) (Capricorn Scientific, Ebsdorfergrund, Germany). Calu-3 cells (EP-CL-0054) were purchased from Elabscience (Houston, TX, USA), and maintained in Eagle’s minimal essential medium (MEM, GlutaMAX^TM^ Supplement) (Thermo Fisher Scientific, Carlsbad, CA, USA), supplemented with 10% FBS, 0.1 mM non-essential amino acids, and 1 mM sodium pyruvate. BHK21/G43 (Rentsch and Zimmer, 2011) were kindly provided by S. Pöhlmann (Leibniz Institute for Primate Research, Göttingen, Germany), and maintained in DMEM supplemented with 10% FBS. Expression plasmids (2 μg, unless otherwise specified) were introduced into HEK293T or VeroE6 cells by transfection for 24 h with the use of Polyethylenimine “Max” (PEI MAX; Polysciences, Warrington, PA, USA). The absence of mycoplasma contamination was confirmed with the use of a PCR Mycoplasma Test Kit (Takara, Kusatsu, Japan).

### Reagents and antibodies

Antibodies to SARS-CoV S protein (#40150-R007), to RFP (#PM005), to VSV G protein (#EB0010), and to VSV M protein (#MABF2347) were obtained from Sino Biological (Beijing, China), Medical & Biological Laboratory (Nagoya, Japan), Kerafast (Boston, MA, USA), and Merck (Darmstadt, Germany), respectively. Hoechst 33342, carbocyanine dyes (DiO, DiI, and DiD), and Alexa Fluor 647– or Alexa Fluor 488–labeled antibodies to rabbit or mouse immunoglobulin G were obtained from Thermo Fisher Scientific. Horseradish peroxidase (HRP)–conjugated goat secondary antibodies were obtained from Jackson ImmunoResearch Laboratories (West Grove, PA, USA). Trypsin was obtained from FujiFilm-Wako (Osaka, Japan), and cell-dissociation solutions were from Thermo Fisher Scientific and Biological Industries (Beit-Haemek, Israel). Mifepristone, camostat mesylate, and E-64d were obtained from Sigma-Aldrich. Nafamostat mesylate was obtained from TCI (Tokyo, Japan).

### Plasmids

Expression vectors for the S protein of SARS-CoV or SARS-CoV-2 (pCG1-SARS-CoV-S and pCG1-SARS-CoV-2-S) (Hoffmann *et al.*, 2020) were kindly provided by S. Pöhlmann. The coding sequence for mCherry was cleaved out of pFX-Tom20-mCherry (Kashiwagi *et al.*, 2019b) by digestion with *Bam*HI and *Bgl*II, and was then subcloned into the *Bam*HI sites of the expression vectors for the S proteins to obtain pCG1-mCherry-SARS-CoV-S and pCG1-mCherry-SARS-CoV-2-S. The pBABE-puro plasmid encoding a puromycin resistance gene was obtained from Addgene.

### Generation of stable cell lines

pCG1-mCherry-SARS-CoV-S or pCG1-mCherry-SARS-CoV-2-S was linearized with *Puv*I and introduced together with pBABE-puro into HEK293T or VeroE6 cells by transfection for 24 h with the use of PEI MAX. The cells were then cultured in DMEM supplemented with 10% FBS and puromycin (10 μg/ml), and puromycin-resistant colonies were collectively harvested. Expression of the mCherry-tagged S proteins was confirmed by immunofluorescence analysis.

BEAS-2B cells stably expressing human ACE2 were generated by lentivirus-mediated gene transfer. HEK293T cells were thus transfected with pLVX-ACE2-IRES-BLD (Daly *et al.*, 2020), pCAG-HIVgp, and pCMV-VSVG-RSV-Rev (Miyoshi *et al.*, 1998) for 48 h, after which the culture supernatant was collected. BEAS-2B cells were exposed to the recombinant lentivirus–containing supernatant for 1 h at 35°C, cultured for 2 days at 35°C in DMEM supplemented with 10% FBS, and further cultured for 14 days in DMEM supplemented with 10% FBS and blasticidin (20 μg/ml) (Funakoshi, Tokyo, Japan). The resulting blasticidin-resistant colonies were collectively harvested, and the constituent cells were maintained in DMEM supplemented with 10% FBS and blasticidin (15 μg/ml).

### Preparation of VSV pseudoviruses

Pseudotyped viruses were generated according to an established protocol (Hoffmann *et al.*, 2020) or its modified version developed in the present study. VSVΔG-G, a replication-defective recombinant VSV, was kindly gifted from S. Pöhlmann. The glycoprotein (G) gene in its genome is substituted by an expression cassette for enhanced GFP (EGFP), which requires an exogenous supply of envelope proteins to produce infectious pseudovirus particles. To propagate VSVΔG-G, BHK21/G43 cells (which stably express VSV G protein) were incubated in the presence of mifepristone (10 nM) for 6 h and then exposed to VSVΔG-G at a multiplicity of infection (MOI) of 1 focus-forming unit (FFU) per cell for 1 h at 35°C. After the inoculum was removed, the cells were further incubated for 15 h at 35°C in fresh DMEM. The resulting supernatant was centrifuged to remove cellular debris and stored as VSVΔG-G suspension at –80°C until use.

For generation of VSVΔG-S particles, cells expressing S protein of SARS-CoV or SARS-CoV-2 (either stably or transiently) were exposed to VSVΔG-G at an MOI of 3 FFU per cell for 1 h at 35°C. After removing the inoculum, the cells were further incubated with fresh medium in the presence of antibodies to VSV G for 15 h in order to neutralize the parental viruses. The culture supernatants were passed through a filter (pore size of 0.45 μm) and subjected to ultracentrifugation, and the virus pellets were resuspended in phenol red-free DMEM/F12 (Thermo Fisher Scientific) and stored at –80°C until use.

To determine the FFU of the pseudoviruses, VeroE6 cells (VSVΔG-G) or BEAS-2B cells expressing ACE2 (VSVΔG-S) were incubated with the virus suspensions for 1 h (VSVΔG-G) or 2 h (VSVΔG-S) at 35°C. After removing the inoculum, the cells were cultured in fresh DMEM for 15 h (VSVΔG-G) or 14 h (VSVΔG-S) at 35°C before staining with Hoechst 33342 for 15 min. The FFU value of the pseudovirus suspension was determined by counting the number of GFP-positive cells under a fluorescence microscope with the use of the “Multi-wavelength cell scoring” module of MetaMorph software (Molecular Devices, San Jose, CA, USA).

### Fluorescence microscopy

Fluorescence imaging and data analysis were performed essentially as described previously (Fujioka *et al.*, 2019; Kashiwagi *et al.*, 2019a). In brief, cells transfected with mCherry-CoV-S or mCherry-CoV-2-S vectors were stained with Hoechst 33342 for 15 min and placed in a stage-top incubation chamber maintained at 37°C on a Nikon Eclipse T*i*2 microscope (Nikon, Tokyo, Japan) equipped with a Zyla5.5 scientific complementary metal oxide semiconductor camera (Oxford Instruments, Belfast, UK), PlanApo 4×/0.2 and 10×/0.45 objective lenses, a TI2-CTRE microscope controller (Nikon), a TI2-S-SE-E motorized stage (Nikon). The cells were illuminated with an X-Cite turbo system (Excelitas Technologies, Waltham, MA, USA). The sets of excitation and emission filters and dichroic mirrors adopted for this observation included GFP HQ (Nikon) for EGFP, Cy3 HQ (Nikon) for mCherry, or DAPI-U HQ (Nikon) for Hoechst 33342. For live-cell imaging, cells were incubated at 37°C with a Chamlide incubator system (Live Cell Instrument, Seoul, Korea).

### Fluorescent labeling of pseudoviruses

Fluorescently labeled pseudoviruses were prepared with the use of red fluorescent lipophilic carbocyanine dyes (DiI and DiD) or a green fluorescent dye (DiO). Pseudovirus suspension (1 ml) was incubated for 1 h at room temperature with 6 μl of 100 μM DiD, DiI, or DiO stock solution in the dark (Fujioka *et al.*, 2018; Nanbo *et al.*, 2010). DiI and DiD were mainly used for single-color labeling, while DiO was used only for two-color labeling with a red fluorescent protein, mCherry. The stained particles were adsorbed to a PEI MAX–coated 96-well glass-based plate, fixed with 3% paraformaldehyde for 15 min at room temperature, and incubated with 1% bovine serum albumin. They were further incubated overnight at 4°C with a rabbit monoclonal antibody to SARS-CoV S protein (1:1000 dilution), after which immune complexes were detected by incubation for 1 h at room temperature in the dark with Alexa Fluor 488-conjugated secondary antibodies (1:250 dilution).

### Immunofluorescence analysis with confocal microscopy

BEAS-2B cells expressing ACE2 were incubated with pseudotyped viruses for 1 h at 4°C to allow for virus attachment. The cells were then fixed with 3% paraformaldehyde for 15 min at room temperature and incubated with 1% bovine serum albumin to block nonspecific binding of antibodies. They were further incubated overnight at 4°C with primary antibodies (1:1000 dilution), after which immune complexes were detected by incubation for 1 h at room temperature in the dark with Alexa Fluor 488- or Alexa Fluor 647-conjugated secondary antibodies (1:250 dilution). Nuclei were visualized by staining with Hoechst 33342. Images were acquired with an IX83 microscope (Olympus, Tokyo, Japan) equipped with a BioPoint MAC 6000 filter and shutter control unit (Ludl Electronic Products, Hawthorne, NY, USA), an automated XY-stage (Chuo Precision Industrial, Tokyo, Japan), a UPlanSApo 60×/1.35 oil objective lens, X-lightV3 (CrestOptics, Rome, Italy) spinning-disk confocal unit, and an iXon Ultra 888 electron-multiplying charge-coupled device (EM-CCD) camera (Oxford Instruments, Abingdon-on-Thames, UK). The cells were illuminated with an LDI laser light source (Chroma Technology Corp., Bellows Falls, VT, USA) through a ZET405/488/561/640x excitation filter (Chroma Technology Corp.). Emission filters adopted for these observations included ET440/40m for Hoechst 33342, ET525/50m for EGFP and Alexa Fluor 488, ET600/50m for mCherry and Alexa Fluor 594, and ET700/75m for Alexa Fluor 647. A ZT405/488/555/640 dichroic mirror (Chroma Technology Corp.) was used throughout this observation. MetaMorph software (Molecular Devices) was used for the control of microscopes and peripheral equipment.

### Immunoblot analysis

Transfected HEK293T cells were lysed in a lysis buffer [50 mM Tris-HCl (pH 7.4), 150 mM NaCl, 1%Nonidet P-40, 0.5% sodium deoxycholate, 0.1% SDS, 1 mM Na_3_VO_4_, cOmplete Protease Inhibitor Cocktail (Sigma-Aldrich)] for 30 min on ice. The lysates were centrifuged at 20,000×*g* for 10 min at 4°C, and the resulting supernatants were subjected to SDS-polyacrylamide gel electrophoresis. The separated proteins were transferred to a polyvinylidene difluoride membrane (Bio-Rad, Hercules, CA, USA) and subjected to immunoblot analysis. Immune complexes were detected with HRP-conjugated secondary antibodies, ECL Western Blotting Detection Reagent (Cytiva, Tokyo, Japan) and a MIIS imaging system (Givetechs, Sakura, Japan).

### Flow cytometry

Pseudotyped viruses were incubated at 4°C for 12 h with anti-SARS-CoV-2 S and anti-VSV M antibodies (1:1000 dilution) and were further incubated for 1 h at room temperature with an Alexa Fluor 488- or an Alexa Fluor 647-conjugated secondary antibody (1:250 dilution). Flow cytometry analysis was performed on a FACSAriaII cell sorter (BD Bioscience, San Diego, CA, USA), and data were analyzed with FACSDiva software (BD Bioscience, version 6).

### Quantification and statistical analysis

Quantitative data are presented as means+SEM from at least three independent experiments and were compared with Student’s *t*-test (parametric test between two conditions) or by one-way analysis of variance (ANOVA) followed by the Tukey honestly significant difference (HSD) post hoc test (among multiple conditions). No statistical methods were applied to predetermine sample size. Experiments were performed unblinded. A *p* value of <0.05 was considered statistically significant, and all statistical analysis was performed with JMP Pro software (SAS Institute Inc., Cary, NC, USA, version 15.0.0).

## Results and Discussion

**Fig. 1:**
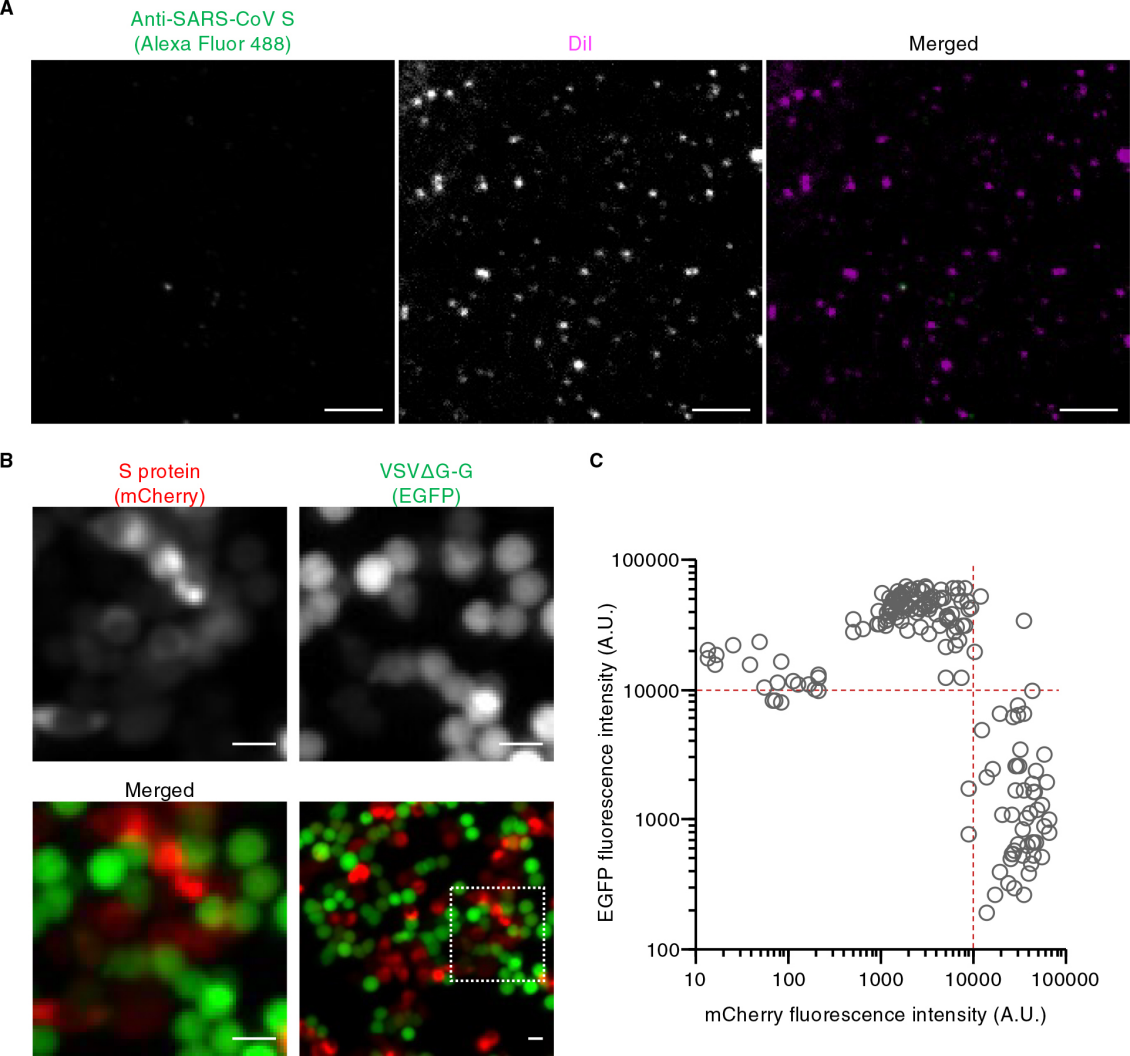
Pseudotyped virus particles produced from cells transiently expressing SARS-CoV S protein are not loaded with S protein (A) HEK293T cells were transfected with an expression vector for SARS-CoV S protein as well as infected with VSVΔG-G. The culture supernatant was collected and stained with DiI as well as subjected to immunofluorescence staining with antibodies to SARS-CoV S protein and Alexa Fluor 488–conjugated secondary antibodies. The virus particles were then adsorbed onto a polyethylenimine-coated glass-based plate and observed with a confocal fluorescence microscope. Representative images are shown. Bars, 10 μm. (B) HEK293T cells were transfected with an expression vector for mCherry-tagged SARS-CoV S protein and infected with VSVΔG-G for 16 h. They were then observed with a fluorescence microscope for detection of mCherry (red) and GFP (green) fluorescence. Representative images are shown. The boxed region in the lower right panel is shown at higher magnification in the other panels. Bars, 10 μm. (C) Fluorescence intensities of GFP and mCherry in individual cells as in (B). See also Fig. S1.

We initially aimed to evaluate the loading efficiency of S proteins on VSV pseudotyped virus particles. According to the published protocol (Hoffmann *et al.*, 2020), HEK293T cells transiently expressing SARS-CoV S protein were exposed to VSVΔG bearing G protein of VSV (VSVΔG-G), and the resulting culture supernatant was harvested as a pseudovirus suspension in the presence of neutralizing antibodies to VSV G protein (to eliminate parental VSVΔG-G). The pseudotyped virions obtained in this manner were stained with the lipophilic carbocyanine dye DiI (Fig. S1A) and then subjected to immunofluorescence analysis with a rabbit monoclonal antibody to SARS-CoV S protein. Confocal fluorescence imaging revealed that only a small proportion of the obtained particles harbored S protein (Fig. 1A).

To examine S protein expression in the pseudotyped virion-producing cells, we prepared an expression vector for a fluorescent protein-tagged form of the protein. The coding sequence for mCherry, a red fluorescent protein (RFP), was thus inserted upstream of the NH_2_-terminal signal sequence of S protein, with the expectation that the fluorescent tag would be cleaved together with the signal sequence in the ER and so would not suppress virion formation. Indeed, immunoblot analysis of HEK293T cells transfected with the vector for mCherry-tagged S protein revealed separate fragments corresponding to mCherry and to S protein (Fig. S1B), and no red fluorescence signal was detected from the VSVΔG-mCherry-S pseudoparticles produced by these cells (Fig. S1C). Immunofluorescence analysis also demonstrated different subcellular localization of mCherry and S protein in mCherry-S expressing cells (Fig. S1D). Furthermore, determination of focus-forming units (FFU) by counting the number of cells positive for green fluorescent protein (GFP) encoded by the reporter gene packaged in the genome of parental VSVΔG did not reveal a marked difference in infectivity between VSVΔG-mCherry-S and VSVΔG-S pseudoviruses in BEAS-2B cells expressing ACE2, also a receptor for SARS-CoV (Fig. S1E). This result also supported the notion that the chimeric protein undergoes proteolytic cleavage and does not inhibit particle formation.

Fluorescence imaging of HEK293T cells transfected with expression vectors for mCherry-S or mCherry showed that mCherry-S expression interfered with VSVΔG-G infection; the GFP signal was thus detected in cells with no or only a low level of mCherry-S expression but not in highly red fluorescent cells (Fig. 1B). Quantitative analysis revealed that GFP-positive cells were indeed highly enriched in cells in which red fluorescence intensity was <10,000 arbitrary units (A.U.) (Fig. 1C), whereas a high level of mCherry expression only partially suppressed VSVΔG-G infection (Fig. S1F). These observations also suggested that virus particles lacking envelope protein might be produced from cells with no mCherry-S expression. Indeed, immunofluorescence analysis with antibodies to VSV M protein showed that the supernatant of vector-transfected, VSVΔG-G-exposed HEK293T cells contained fluorescent puncta even when the vector did not encode an envelope protein (Fig. S1G).

**Fig. 2:**
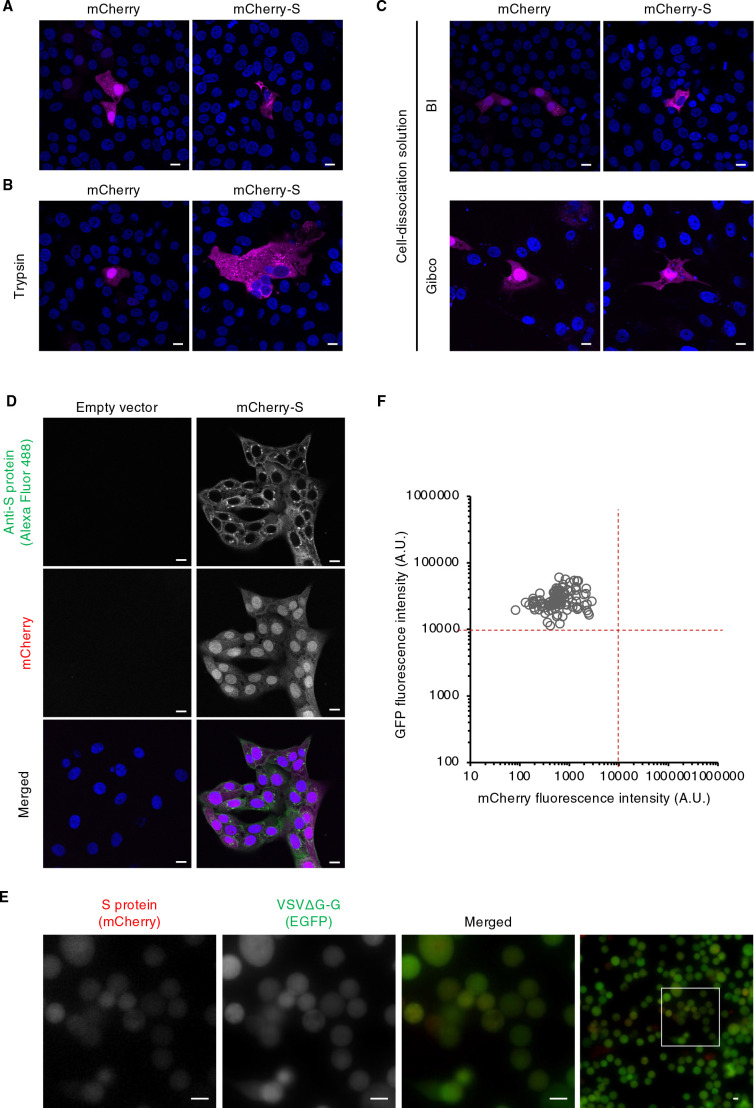
Establishment of cell lines stably expressing S protein (A) VeroE6 cells transfected with an expression vector for mCherry or mCherry-tagged S protein of SARS-CoV (mCherry-S) that had been linearized with *Puv*I were stained with Hoechst 33342 and observed by fluorescence microscopy. Representative images of mCherry (magenta) and Hoechst 33342 (blue) fluorescence are shown. Bars, 10 μm. (B and C) Cells prepared as in (A) were harvested by exposure to trypsin (B) or to enzyme-free cell-dissociation solutions from Biological Industries (BI) or Gibco (C). They were then transferred to cell culture dishes and cultured for 24 h before fluorescence microscopic analysis as in (A). Representative images are shown. Bars, 10 μm. (D) Cells transfected with the mCherry-S vector (or the corresponding empty vector) as in (A) were cultured in the presence of puromycin for 14 days, stained with Hoechst 33342, and subjected to immunofluorescence analysis with antibodies to SARS-CoV S protein and Alexa Fluor 488–conjugated secondary antibodies. Representative fluorescence microscopic images of S protein immunostaining (green) as well as of mCherry (magenta) and Hoechst 33342 (blue) fluorescence are shown. Bars, 10 μm. (E) VeroE6 cells stably expressing mCherry-tagged S protein were exposed to VSVΔG-G for 16 h and then observed with a fluorescence microscope. Representative images of EGFP and mCherry fluorescence and their merged images are shown. The boxed regions in the right panels are shown at higher magnification in the left panels. Bars, 10 μm. (F) Scatter plot for the fluorescence intensities of EGFP and mCherry (mCherry-tagged S protein) in individual stably transfected VeroE6 cells treated as in (E). See also Fig. S2.

Our results together suggested that a low and persistent level of S protein expression might be required for the production of pseudotyped virus particles that uniformly bear S protein. Indeed, transfection of less expression vector for S protein resulted in lower S protein expression (Fig. S1H) and subsequent higher viral production (Fig. S1I). We therefore attempted to establish cell lines that stably express S protein at a low level. However, although HEK293T cells are used in the standard protocol for VSV-based pseudotyped virus production, we found that HEK293T cells expressing mCherry-S manifested a shrunken morphology compared with those expressing mCherry alone (Fig. S1J). We next introduced the expression vector for mCherry-S into VeroE6 cells, which have been widely adopted for SARS-CoV research. Fluorescence imaging showed that the morphology of VeroE6 cells expressing mCherry-S was similar to that of those expressing mCherry alone (Fig. 2A). To generate stable cell lines, we isolated transfected VeroE6 cells by treatment with trypsin and subjected them to puromycin selection. After incubation for 24 h, multinucleated cells, indicative of cell-cell fusion resulting from S protein activation by trypsin cleavage, were apparent for the cells expressing mCherry-S (Fig. 2B). To avoid cleavage of S protein, we used enzyme-free cell-dissociation solutions for cell isolation. Treatment with such solutions did not induce cleavage of S protein (Fig. S2) or multinucleated cells (Fig. 2C), thus allowing the generation of cell lines stably expressing S protein (Fig. 2D).

**Fig. 3:**
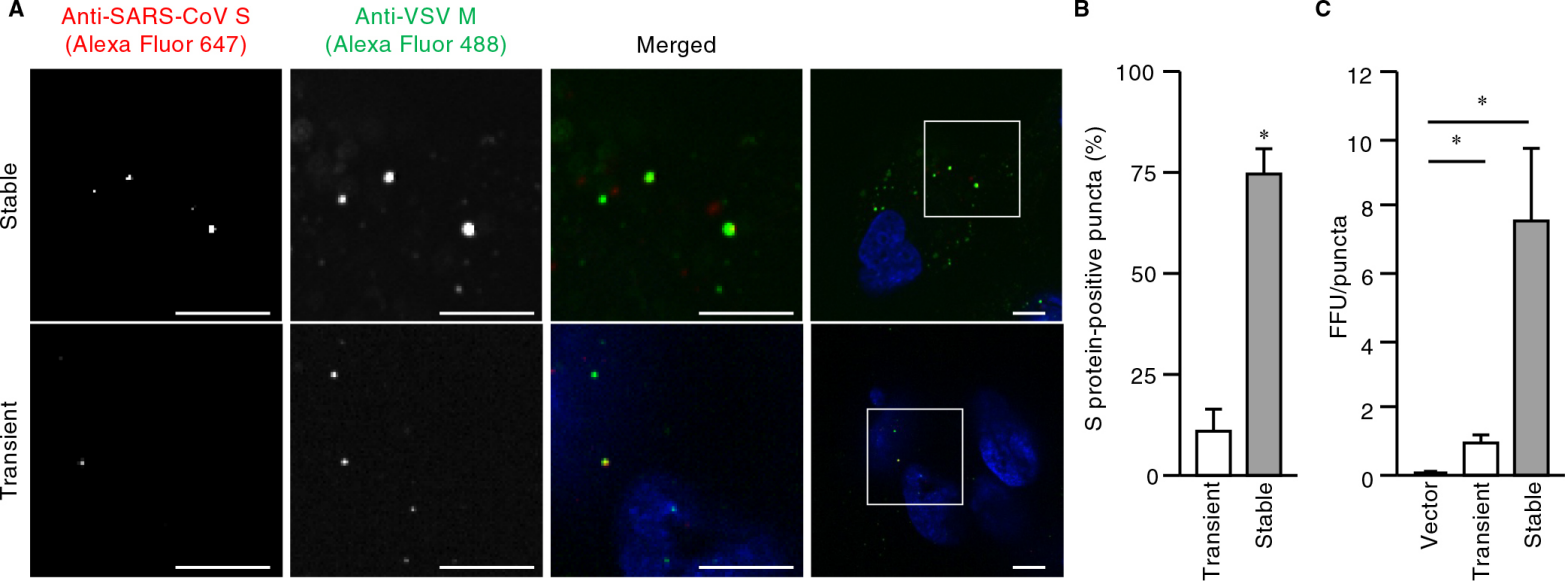
A large proportion of pseudotyped virus particles produced from VeroE6 cells stably expressing SARS-CoV S protein are loaded with S protein. (A) VeroE6 cells stably expressing or HEK293T cells transiently expressing SARS-CoV S protein were infected with VSVΔG-G for 16 h, and equal numbers of pseudoviruses released into the culture supernatants were added to ACE2-expressing BEAS-2B cells for 1 h at 4°C. The latter cells were then fixed, stained with Hoechst 33342 (blue), subjected to immunofluorescence analysis with antibodies to SARS-CoV S protein and VSV M protein, and observed with a confocal microscope. Representative images are shown. The boxed regions in the right panels are shown at higher magnification in the panels to the left. Bars, 10 μm. (B) Quantification of the fraction of S protein-positive puncta among all M protein–positive puncta for images as in (A). Data are means+SEM from three independent experiments. *, *p*<0.001 (Student’s *t*-test). (C) BEAS-2B cells stably expressing ACE2 were exposed to pseudotyped viruses prepared as in (A) for determination of the number of GFP-positive cells and calculation of FFU of virus suspension. FFU was normalized by the amount of pseudotyped viruses, which was determined by the area of puncta of DiD-stained viruses on a polyethylenimine-coated glass-based plate. Data are means+SEM from three independent experiments. *, *p*<0.004 (one-way ANOVA with Tukey’s HSD post hoc test). See also Fig. S3.

We next examined whether such a cell line was susceptible to VSVΔG-G infection for production of pseudoviruses harboring S protein. Fluorescence microscopy and quantitative analysis of the virus-exposed cells revealed that the mCherry fluorescence intensity of all cells was <10,000 A.U. (the desired upper limit as determined in Fig. 1C), and that most cells were positive for GFP, indicative of uniform VSVΔG-G infection (Fig. 2E, F). The virus particles produced by the cells were then stained with DiD and adsorbed on a polyethylenimine-coated glass-based dish to determine the amount of particles per unit volume by measurement of the area of DiD-positive puncta with a confocal microscope. The amount of particles produced by VeroE6 cells stably expressing mCherry-S (1.3×10^4^ area of puncta (AOP)/μl) was ~10% of that of those produced by HEK293T cells transiently expressing mCherry-S (1.3×10^3^ AOP/μl) (Fig. S3A, B). BEAS-2B cells expressing ACE2 were then exposed to the pseudoviruses for 1 h at 4°C (to allow for virus attachment) and subjected to immunofluorescence analysis with antibodies to SARS-CoV S protein and to VSV M protein in order to allow visualization of S protein and all pseudovirus particles, respectively. Confocal imaging showed that, in contrast to the virus particles produced by transiently transfected HEK293T cells, a large proportion of virus particles produced by the stably transfected VeroE6 cell line were positive for S protein (Fig. 3A). Indeed, quantitative analysis with the use of microscopy and flow cytometry revealed that the fraction of S protein-positive particles generated by the stable cell line was about seven times that generated by the transiently transfected cells (Fig. 3B and Fig. S3D, E). These results thus demonstrated the efficient production of pseudoviruses that were essentially all loaded with S protein by the stable cell line.

We further examined whether the titer of pseudovirus particles generated by the stable cell line was higher than that of particles generated by the conventional method. There was no significant difference between the FFU values obtained for the two types of pseudoviruses (Fig. S3C). To normalize infectivity, we prepared dilutions of the virus suspensions (down to 1.0 FFU/μl) and plotted FFU against the area of puncta. A linear relation was apparent for each type of pseudovirus, although the slopes of the regression lines differed (Fig. S3F). The FFU value normalized by the amount of viruses produced by the stable cell line was 6.4 times the corresponding value for the viruses produced by the transiently transfected cells (Fig. 3C), indicating that the modified method established in this study results in a substantial improvement in infectious pseudovirus particle production. In addition, the titer of pseudoviruses generated by the stable VeroE6 cell line was higher than that of particles generated by transiently transfected VeroE6 cells (Fig. S3G), suggesting that such high efficiency is caused by the expression system, but not cell context.

**Fig. 4:**
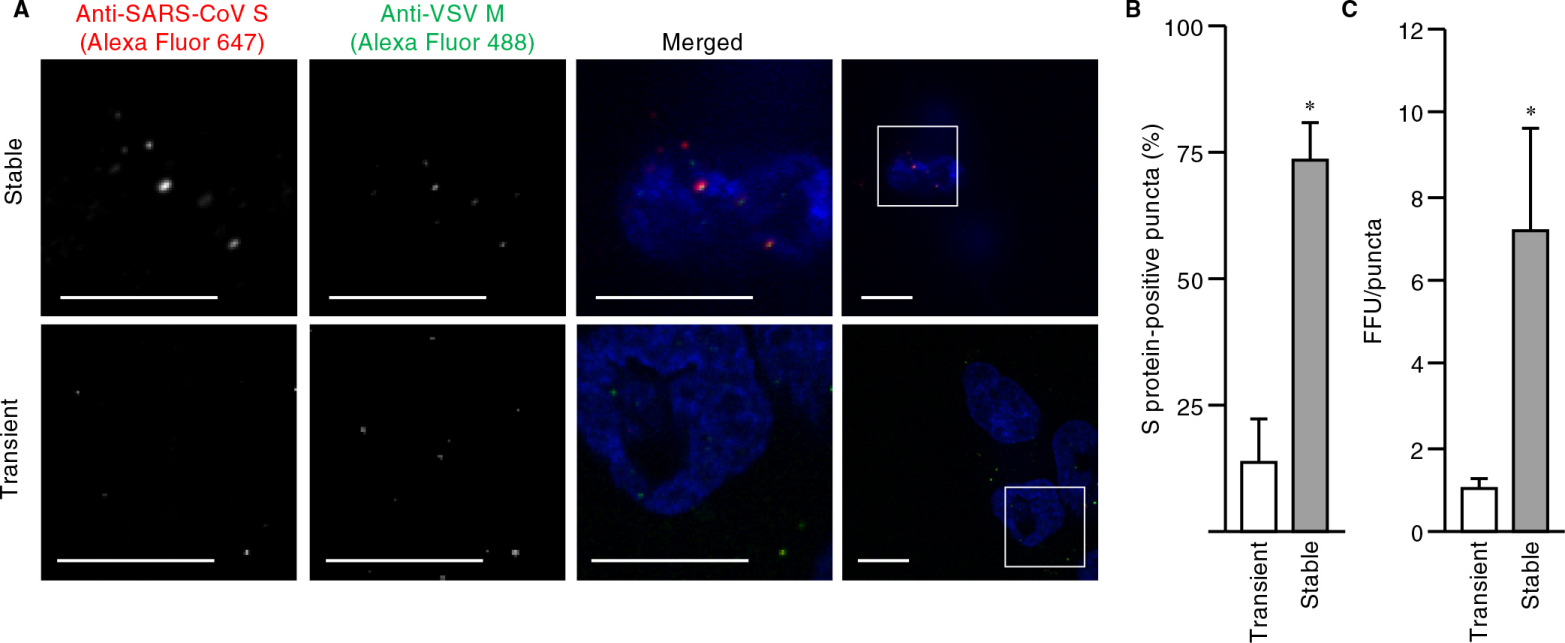
Production of pseudoviruses loaded with S protein of SARS-CoV-2 by a stable cell line (A) BEAS-2B cells expressing ACE2 were exposed for 1 h at 4°C to pseudotyped viruses produced from a VeroE6 cell line stably expressing or HEK293T cells transiently expressing S protein of SARS-CoV-2. The cells were then fixed, stained with Hoechst 33342 (blue), subjected to immunofluorescence staining with antibodies to SARS-CoV S protein and VSV M protein, and examined with a confocal microscope. Representative images are shown. The boxed regions in the bottom panels are shown at higher magnification in the panels above. Bars, 10 μm. (B) Quantification of the fraction of S protein-positive puncta among all M protein–positive puncta in images as in (A). Data are means+SEM from three independent experiments. *, *p*<0.001 (Student’s *t*-test). (C) FFU normalized by the area of puncta for the pseudoviruses produced as in (A) was determined as in Fig. S4. Data are means+SEM from three independent experiments. *, *p*<0.004 (one-way ANOVA with Tukey’s HSD post-hoc test). See also Fig. S4.

Finally, we attempted to produce pseudotyped virus particles with envelopes bearing the S protein of SARS-CoV-2 by our newly established protocol. We thus established a VeroE6 cell line that stably expresses SARS-CoV-2 S protein (Fig. S4A). The fraction of SARS-CoV-2 S protein-positive puncta (Fig. 4A, B) and FFU normalized by the area of DiD-positive puncta (Fig. 4C and Fig. S4B, C) were five and seven times as high, respectively, for viruses produced from the VeroE6 cell line as were those for viruses produced from transiently transfected HEK293T cells. Furthermore, to evaluate whether the pseudoviruses produced by stable cell lines mimic authentic SARS-CoV-2 entry, we performed viral entry assay with the use of inhibitors for membrane fusion (camostat and nafamostat) and endocytosis (E-64d) (Hoffmann *et al.*, 2020; Kawase *et al.*, 2012; Kim *et al.*, 1995; Yamamoto *et al.*, 2016). In Calu-3 cells, which SARS-CoV-2 infects via membrane fusion (Hoffmann *et al.*, 2020), camostat and nafamostat suppressed the viral infection (Fig. S4D). By contrast, all the inhibitors partially suppressed the infection in VeroE6/TMPRSS2 cells (Fig. S4E). These results are consistent with previous reports (Hoffmann *et al.*, 2020; Shang *et al.*, 2020) and ensure the proper entry mode of the pseudoviruses produced in this study. Our procedure was thus shown to be highly efficient with regard to pseudovirus production for studies of the entry of SARS-CoV or SARS-CoV-2.

In this study, we unexpectedly found that a large proportion of pseudotyped virus particles produced by the conventional protocol (Hoffmann *et al.*, 2020) did not bear coronavirus S proteins. This finding raised the concern that S protein–free virions might affect the entry process of, or cellular responses to, S protein–bearing particles and thereby confound experimental outcomes. We therefore established a modified protocol based on stable cell lines in order to generate pseudoviruses for which a large proportion of the virus particles harbor heterologous envelope proteins. Alternatively, it has been attempted to improve the infectivity of the pseudoviruses by engineering the S protein. Recent studies have reported that the deletion of the C-terminal region of S protein facilitates its loading into pseudovirus particles by increasing the efficiency of the plasma membrane localization of S protein in virus-producing cells (Johnson *et al.*, 2020; Yu *et al.*, 2021). In addition, it has also been reported that D614G point mutation brings about resistance to proteolytic cleavage during S protein production, subsequently resulting in higher S protein incorporation into the virion (Zhang *et al.*, 2020). Our method can easily incorporate such new knowledge regarding S protein by generating stable cell lines, facilitating higher titer viruses. Other alternative methods that are free of such issues relating to virus particles lacking envelope protein, including reverse genetic systems for in vitro virus assembly from SARS-CoV-2 full-length cDNA (Torii *et al.*, 2021; Xie *et al.*, 2020), have been developed. However, given that the VSV-based pseudotyped virus systems have been adopted by many researchers, the modification described here should be readily incorporated into current protocols and lead both to a better understanding of virus entry and to accelerated vaccine and therapeutic development for SARS-CoV-2.

## Conflict of interest

Y.F. and Y.O. have a patent pending that includes claims relating to the present study. Other authors declare no competing interests.
