# Supplementary material for: A method for the generation of pseudovirus particles bearing SARS coronavirus spike protein in high yields
Source: Cell Struct Funct. 2022 Apr 28;47(1):43–53. doi: 10.1247/csf.21047 (PMC10511058; doi:10.1247/csf.21047)
Supplement: Supplementary file 1 — Fig. S1 [file csf_47_21047_1.pdf]

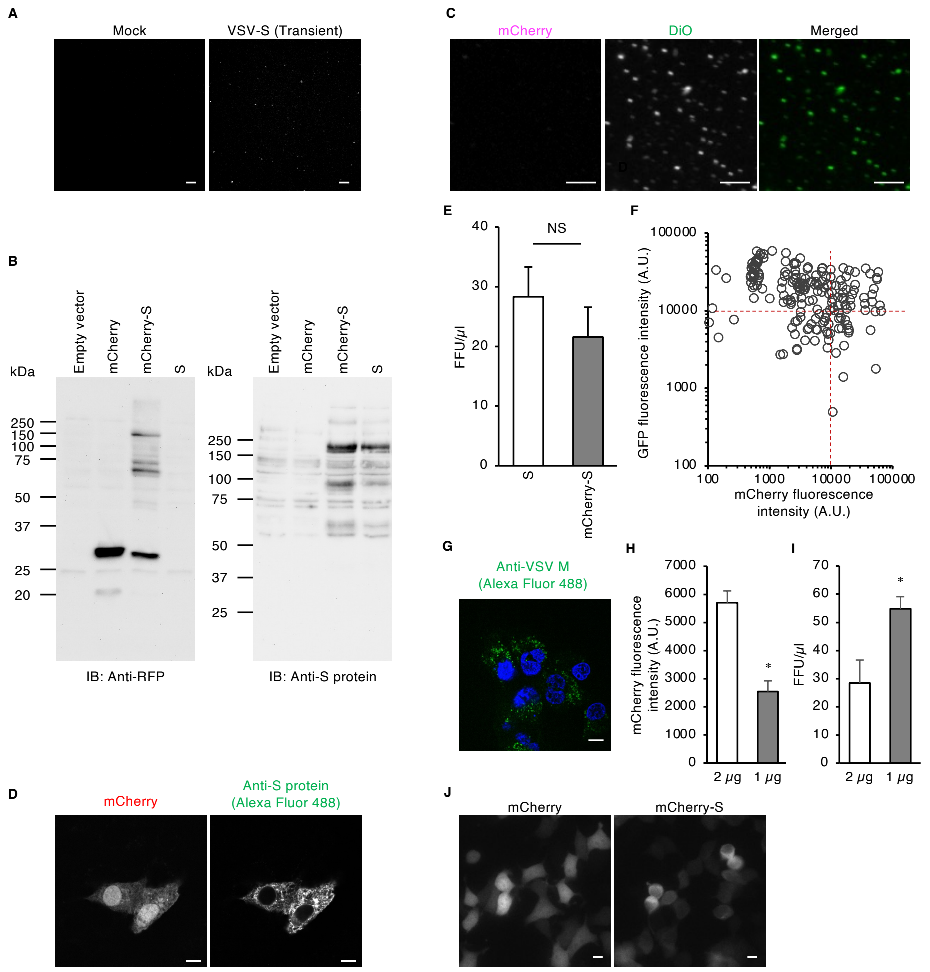

Figure S1. Tagging of SARS-CoV S protein with mCherry does not affect pseudovirus production, related to Figure 1

(**A**) HEK293T cells were transfected with an expression vector for mCherry-S or mCherry (control) for 24 h and infected with VSV Δ G-G. Pseudotyped viruses purified from the culture supernatant were stained with DiI, allowed to adhere to a 96-well glass-based plate coated with polyethylenimine, and then observed with a confocal microscope for detection of DiI fluorescence. Representative images are shown. Bars, 10 µm.

(**B**) HEK293T cells transfected with an expression vector for mCherry, SARS-CoV S protein, or mCherry-tagged S protein (mCherry-S) for 24 h were lysed and subjected to immunoblot (IB) analysis with antibodies to RFP (for detection of mCherry) or to S protein. Representative blots are shown. Of note, mCherry is a derivative of an RFP and therefore detectable by an anti-RFP antibody.

(**C**) Pseudotyped viruses produced from HEK293T cells expressing mCherry-S and infected with VSV Δ G-G were stained with DiO, allowed to adhere to a 96-well glass-based plate coated with polyethylenimine, and then observed with a confocal microscope for detection of mCherry and DiO fluorescence. Representative images are shown. Bars, 10 µm.

(**D**) HEK293T cells expressing mCherry-S were fixed and subjected to immunofluorescence analysis with antibodies to SARS-CoV S protein (green fluorescence). Representative images are shown. Bar, 10 µm.

(**E**) BEAS-2B cells expressing ACE2 were exposed for 16 h to pseudotyped viruses produced from VSVΔG-G–infected HEK293T cells expressing S protein or mCherry-S protein. They

were then stained with Hoechst 33342 and observed with a fluorescence microscope. The number of EGFP-positive cells (FFU), per microliter was determined. Data are means + SEM from three independent experiments. NS, not significant (Student's *t* test).

(**F**) HEK293T cells transfected with an expression vector for mCherry and infected with VSVΔG-G for 16 h were observed with a fluorescence microscope, and the fluorescence intensities of EGFP and mCherry in individual cells were measured.

(**G**) BEAS-2B cells expressing ACE2 were exposed to pseudoviruses produced from empty vector-transfected, VSVΔG-G-infected HEK293T cells, fixed, and subjected to immunofluorescence analysis with antibodies to M protein of VSV (green fluorescence). Nuclei were stained with Hoechst 33342 (blue fluorescence). A representative image is shown. Bar, 10 μm.

(**H, I**) HEK293T cells were transfected with 1 μg or 2 μg of an expression vector for mCherry-S, infected with VSVΔG-G, and after 16 h, observed with a fluorescence microscope. Fluorescence intensities of mCherry were plotted (**H**). BEAS-2B cells stably expressing ACE2 were exposed to the pseudotyped viruses purified from the culture supernatants. The number of GFP-positive cells was counted to calculate the FFU of virus suspension (**I**). Data are means + SEM from three independent experiments. *, $p < 0.05$ (Student's *t*-test).

(**J**) HEK293T cells expressing mCherry or mCherry-tagged S protein were observed with a fluorescence microscope. Representative images are shown. Bars, 10 μm.
