# Supplementary material for: A method for the generation of pseudovirus particles bearing SARS coronavirus spike protein in high yields
Source: Cell Struct Funct. 2022 Apr 28;47(1):43–53. doi: 10.1247/csf.21047 (PMC10511058; doi:10.1247/csf.21047)
Supplement: Supplementary file 2 — Fig. S2 [file csf_47_21047_2.pdf]

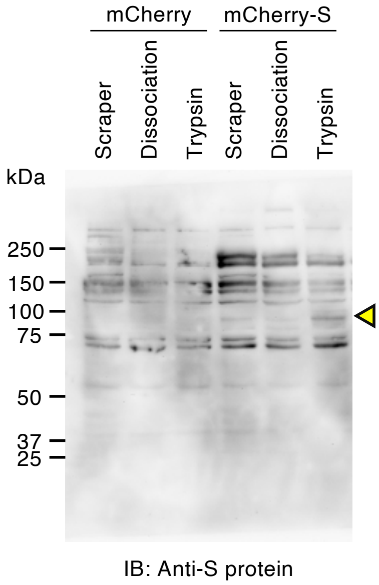

Figure S2. Trypsin-free cell-dissociation solution does not degrade SARS-CoV S protein, related to Figure 2.

VeroE6 cells expressing mCherry or mCherry-tagged SARS-CoV S protein were isolated either by exposure to trypsin or a cell-dissociation solution or with the use of a cell scraper. They were then lysed and subjected to immunoblot analysis with antibodies to SARS-CoV S protein. A representative blot is shown. The arrowhead indicates cleaved S protein.
