# Supplementary material for: A method for the generation of pseudovirus particles bearing SARS coronavirus spike protein in high yields
Source: Cell Struct Funct. 2022 Apr 28;47(1):43–53. doi: 10.1247/csf.21047 (PMC10511058; doi:10.1247/csf.21047)
Supplement: Supplementary file 3 — Fig. S3 [file csf_47_21047_3.pdf]

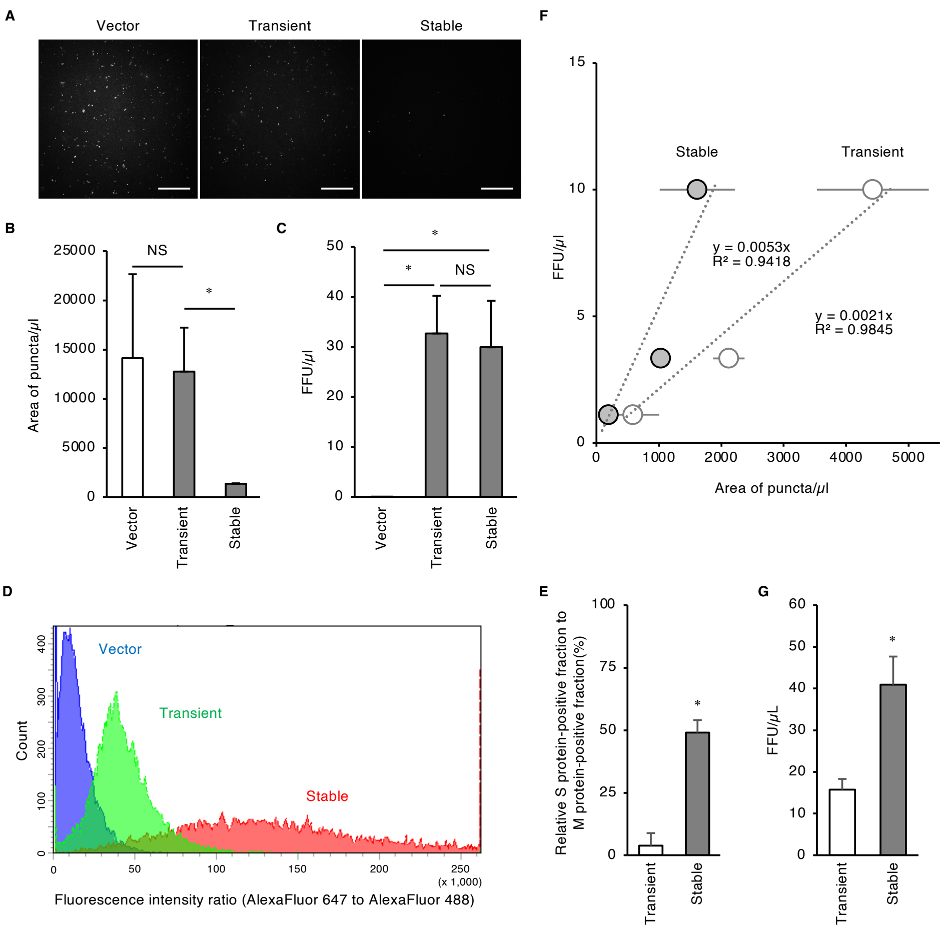

Figure S3. Pseudotyped virus production from VeroE6 cells stably expressing SARS-CoV S protein, related to Figure 3

(**A**) Pseudotyped viruses produced either from HEK293T cells transiently transfected with an expression vector for SARS-CoV S protein (Transient) or with the corresponding empty vector (Vector) or from VeroE6 cells stably expressing SARS-CoV S protein (Stable) were stained with DiD, transferred to a polyethylenimine-coated glass-based plate, and observed with a confocal microscope. Representative images are shown. Bars, 10 μm.

(**B**) Quantification of the area of puncta for images as in (**A**). Data are means + SEM from three independent experiments. NS, not significant; *, $p < 0.001$ (one-way ANOVA with Tukey's HSD post-hoc test).

(**C**) ACE2-expressing BEAS-2B cells were exposed to pseudotyped viruses prepared as in (**A**) for determination of the number of GFP-positive cells and calculation of FFU per microliter of virus suspension. Data are means + SEM from three independent experiments. NS, not significant; *, $p < 0.001$ (one-way ANOVA with Tukey's HSD post-hoc test).

(**D**, **E**) Pseudotyped viruses prepared as in (**A**) were incubated with anti-SARS-CoV S and anti-VSV M antibodies, incubated further for 1 h at room temperature with Alexa Fluor 647- and Alexa Fluor 488-conjugated secondary antibodies, respectively, and subjected to flow cytometry analysis. A representative histogram is shown (**D**). S protein-positive fraction relative to M protein-positive fraction was calculated and plotted (**E**).

(**F**) DiD-labeled VSVΔG-S particles produced by the conventional (transiently transfected HEK293T cells) or modified (stably transfected VeroE6 cells) method were adsorbed onto polyethylenimine-coated plates at various dilutions and the area of puncta was measured with a confocal microscope and plotted against the corresponding FFU values determined as in (**C**). The regression equation and coefficient for each type of virus are indicated.
(**G**) The FFU per microliter of virus suspension was determined as in (**C**) for VSVΔG-S produced either from the VeroE6 cells stably expressing or transiently expressing SARS-CoV S protein. Data are means + SEM from three independent experiments. *, $p < 0.05$ (Student's *t*-test).
