# Supplementary material for: A method for the generation of pseudovirus particles bearing SARS coronavirus spike protein in high yields
Source: Cell Struct Funct. 2022 Apr 28;47(1):43–53. doi: 10.1247/csf.21047 (PMC10511058; doi:10.1247/csf.21047)
Supplement: Supplementary file 4 — Fig. S4 [file csf_47_21047_4.pdf]

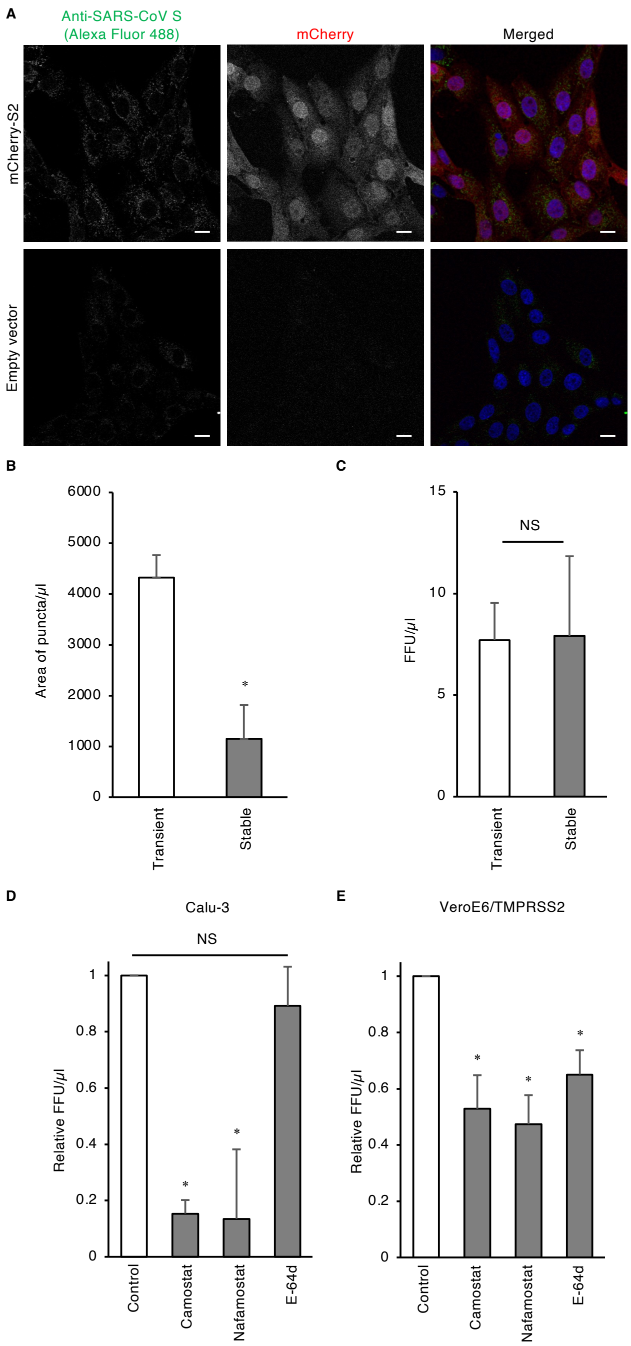

Figure S4. Establishment of a VeroE6 cell line stably expressing S protein of SARS-CoV-2, related to Figure 4

(**A**) VeroE6 cells stably expressing mCherry-tagged S protein of SARS-CoV-2 (mCherry-S2) or parental VeroE6 cells were stained with Hoechst 33342, subjected to immunofluorescence staining with antibodies to SARS-CoV S protein, and examined by fluorescence microscopy. Representative images of S protein (green), Hoechst 33342 (blue), and mCherry (magenta) fluorescence are shown. Bars, 10 μm.

(**B, C**) The area of puncta (**B**) and FFU (**C**) per microliter of virus suspension were determined as in **Figure S3** for VSVΔG-G pseudoviruses produced either from the cell line in (**A**) or from HEK293T cells transiently expressing SARS-CoV-2 S protein. Data are means + SEM from three independent experiments. NS, not significant; *, $p < 0.02$ (Student's *t*-test).

(**D**, **E**) Calu-3 cells (**D**) and VeroE6 cells expressing TMPRSS2 (**E**) were pretreated with camostat, nafamostat, or E-64d for 2 h and exposed to the pseudoviruses for 16 h. The cells were then stained with Hoechst 33342 and observed under a fluorescence microscope to determine the FFU per microliter of virus suspension. Data are means + SEM from three independent experiments. NS, not significant; *, $p < 0.05$ (one-way ANOVA with Tukey's HSD post-hoc test).
